# Effect of a Yeast β-Glucan on the Performance, Intestinal Integrity, and Liver Function of Broiler Chickens Fed a Diet Naturally Contaminated with *Fusarium* Mycotoxins

**DOI:** 10.3390/toxins17020051

**Published:** 2025-01-23

**Authors:** Virginie Marquis, Julie Schulthess, Francesc Molist, Regiane R. Santos

**Affiliations:** 1 Phileo by Lesaffre, 137 Rue Gabriel Péri, 59700 Marcq en Baroeul, France; j.schulthess@phileo.lesaffre.com; 2 Department of Research and Development, Schothorst Feed Research, Meerkoetenweg 26, 8218 NA Lelystad, The Netherlands; fmolist@schothorst.nl (F.M.); rsantos@schothorst.nl (R.R.S.)

**Keywords:** *Fusarium*, mycotoxins, intestine, liver, oxidative stress, inflammation, bacterial translocation, poultry

## Abstract

This study evaluated the effect of a yeast β-glucan on the performance, gut health, liver function, and bacterial translocation of broiler chickens fed a diet contaminated with *Fusarium* mycotoxins. One-day-old male Ross broilers (n = 234) were divided into three treatments with six replicates each, and a cage containing 13 birds was the experimental unit. The animals were fed a maize–soybean-based control diet or maize–soybean diets naturally contaminated with *Fusarium* mycotoxins, where deoxynivalenol (DON) was the major mycotoxin (~3 mg/kg), followed by zearalenone (ZEN) (~0.5 mg/kg). The *Fusarium*-contaminated diet was either supplemented or not with a yeast β-glucan over 28 days. Dietary exposure to *Fusarium* mycotoxins did not affect production performance. On the other hand, *Fusarium* mycotoxin exposure significantly decreased jejunum villus height (VH) and crypt depth (CD) on d13, and this effect was counteracted by the yeast β-glucan. On d28, the jejunum VH:CD ratio was significantly higher in the broiler chickens that were fed the *Fusarium*-contaminated diet with yeast β-glucan (125 mg/kg diet) added to it. The ileal villus area was significantly decreased in the broiler chickens fed *Fusarium*-contaminated diet, regardless of the supplementation with yeast β-glucan. Dietary contamination caused intestinal oxidative stress and inflammation, probably affecting nutrient absorption on d28, and resulted in a significant increase in the translocation of *Escherichia coli* to the liver. Dietary supplementation with yeast β-glucan minimized these negative effects.

## 1. Introduction

Poultry diet contamination with mycotoxins is an unavoidable problem. Among the several mycotoxins that may be found in the final feed of broiler chickens, *Fusarium* mycotoxins such as deoxynivalenol (DON), zearalenone (ZEN), and fumonisin (FUM) are frequently detected [1,2,3]. Despite the uncommon clinical signs associated with these mycotoxins, most studies on broiler chickens have focused on how chronic exposure to low levels of DON results in impaired growth performance [1,4,5], subclinical alterations [6], economic losses, and a negative impact on animal welfare.

Exposure to a wheat-based diet containing 2.3 mg/kg DON did not increase mortality, but the broiler chickens were 100 g lighter than those fed a 0.9 mg/kg DON diet [5]. Exposure to DON impairs intestinal function due to an increase in apoptosis as well as decreases in enterocyte proliferation [7], weakened tight junctions [8,9], and bacterial translocation to the liver [10]. Intestinal damage is characterized by morphologic alterations like decreased villus height [5,11], which results in a diminished nutrient absorption area and subsequently requires energy during intestinal morphological and functional adaptation during chronic exposure [12]. Damage to the tight junctions favors increases intestinal permeability and the risk of the translocation of enteric microorganisms like *Escherichia coli* [10] and *Enterobacteriaceae* in general, as these bacteria naturally belong to the intestinal microbiota. In addition, the suboptimally absorbed nutrients are used as a substrate for pathogenic microorganisms and may induce secondary diseases like subclinical necrotic enteritis [13].

Unfortunately, dietary contamination with a single mycotoxin is a rare event; the rule is multi-contamination with several mycotoxins [14]. Diets naturally contaminated with *Fusarium* mycotoxins commonly present not only DON but also its acetylated forms 3- and 15-Acetyl-DON (3- and 15-ADON) as well as DON-3-glucoside (DON-3G) [6]. Other *Fusarium* mycotoxins commonly found in poultry diets are FUM [2] and ZEN [3].

Broiler chickens fed a maize–soybean-based diet artificially contaminated with 1.5 mg/kg DON and 20 mg/kg FUM gained less body weight than expected [15]. However, the tested FUM contamination level was far higher than that observed in practice. Another maize–soybean-based diet containing 3.2–3.9 mg/kg DON and negligible levels of FUM (0.1 mg/kg) increased the feed conversion ratio (FCR) in broiler chickens [16]. Due to a LOAEL of 30 mg/kg BW per day and a NOAEL of 7.5 mg/kg BW per day, and the ability to convert most ingested ZEN into β-zearalenol (5-fold less potent than ZEN), broiler chickens are considered very resistant to this mycotoxin, and the risk of adverse effects is very low in these animals [17].

Preventive measures can decrease feed contamination but are not effective to completely prevent the presence of mycotoxins in animal diets. An approach involves using feed additives that may deactivate mycotoxins, preventing their absorption in the intestine or enhancing gut health during exposure to mitigate the detrimental effects of mycotoxins. To reduce the intestinal and liver injuries caused by *Fusarium* mycotoxins, it is necessary to tackle oxidative stress, inflammation, and support nutrient absorption. Yeast β-glucan, a polysaccharide comprising a β-1,3-linked D-glucopyranosil backbone with β-1,6-linked side chains, has health-promoting functions, such as immune modulation, anti-inflammatory, antioxidative, and antibacterial activities, as well as beneficial effects on intestinal barrier function [18,19,20].

The aim of our study was to assess the effects of 28 days of dietary exposure to *Fusarium* mycotoxins on the production performance, small intestine morphology and function, liver function, and bacterial translocation of broiler chickens. To this end, broiler chickens were fed a control diet that was marginally or naturally *Fusarium*-contaminated, either supplemented or not with yeast β-glucan. Growth performance was assessed on days 13 and 28. Also, intestinal integrity and function were analyzed via histological and qRT-PCR analyses, which were conducted to study if the intestinal morphology was affected and if genes coding for oxidative stress, inflammation response, tight junctions, and nutrient absorption were influenced by DON and the additive intervention. The liver was analyzed for oxidative stress, inflammation, and metabolic function via qRT-PCR, and bacterial translocation to the liver was assessed.

## 2. Results

### 2.1. Production Performance

The average body weight of the birds at the start of the trial was 41.5 g (41.1–41.9 g) for all treatments. No differences in growth performance or mortality were observed (Table 1).

### 2.2. Jejunum and Ileum Morphometry and Morphological Scores

**Figure 1:**
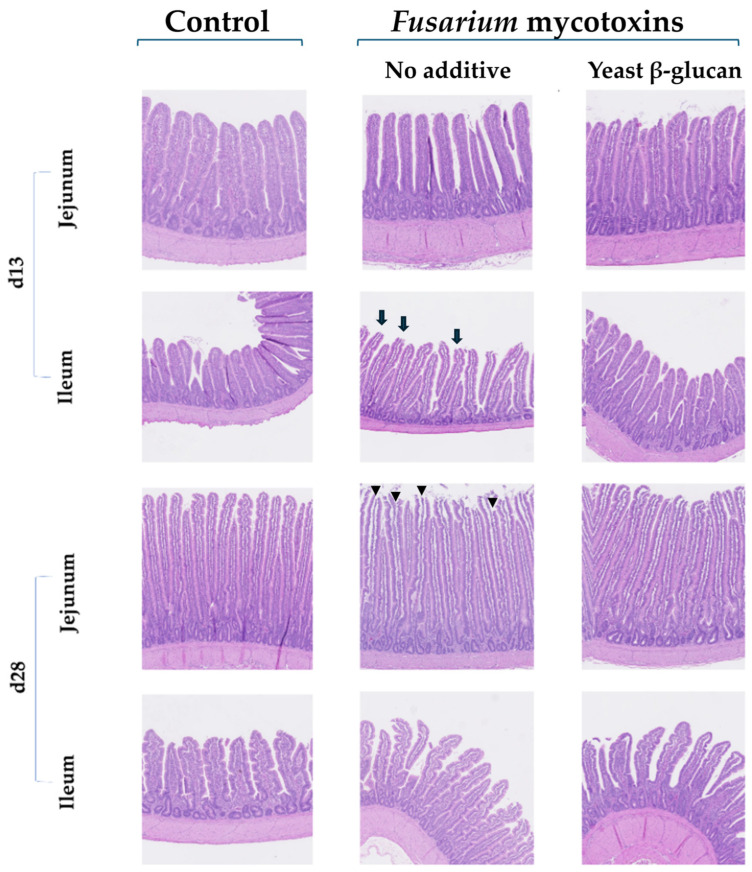
Illustrative images of PAS-hematoxylin-stained sections of jejunum and ileum from broiler chickens fed experimental diets. Arrows indicate damage in villus tips (score 1), and arrowheads indicate extension of subepithelial space with moderate to massive lifting of villi (scores 2 and 3).

The villus height (VH) and crypt depth (CD) of the jejunum of 13-day-old broiler chickens were significantly decreased when the birds were fed the *Fusarium*-contaminated diet. Adding yeast β-glucan to the *Fusarium*-contaminated diet was able to counteract this negative effect. On day 28, the jejunum VH:CD was significantly increased in the broiler chickens fed the *Fusarium*-contaminated diet supplemented with yeast β-glucan. The jejunum morphologic scores remained unaffected (Table 2). No morphometric changes were observed in the ileum, except on day 28, where the villus area was significantly decreased in the ileum of the broiler chickens fed the *Fusarium*-contaminated diet with or without yeast β-glucan. The ileum morphologic scores remained unaffected (Table 3). Illustrative images of the jejunum and ileum from the broiler chickens fed the experimental diets are given in Figure 1.

### 2.3. mRNA Expression of Markers of Gut Integrity and Liver Function

**Figure 2:**
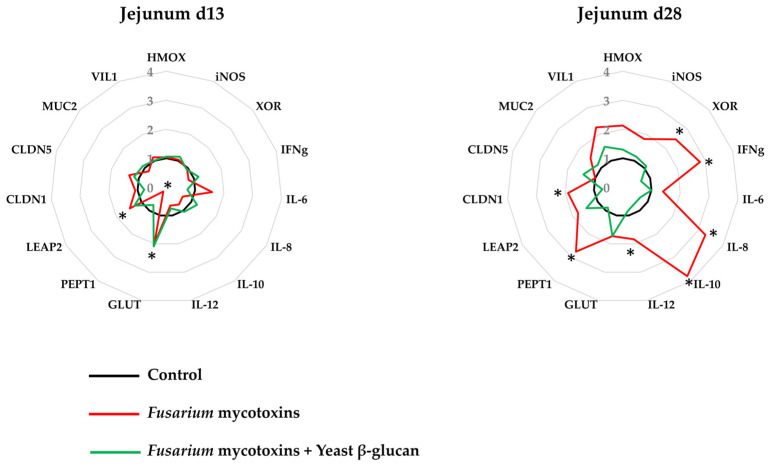
Effect of the experimental diets on the mRNA expression of the markers of oxidative stress, inflammation, nutrient absorption, and gut integrity. * Indicates significant difference from control (*p* < 0.05).

On day 13, a significant upregulation of liver-expressed antimicrobial peptide 2 (LEAP2) and significant downregulation of peptide transporter 1 (PEPT1) were observed in the jejunum of the broiler chickens fed the *Fusarium*-contaminated diet, and this effect was no longer observed when the *Fusarium*-contaminated diet was supplemented with yeast β-glucan. Furthermore, a significant upregulation of glucose transporter 1 (GLUT1) was observed in the broiler chickens fed the *Fusarium*-contaminated diet supplemented with yeast β-glucan (Figure 2).

On day 28, the jejunum of the broiler chickens fed a *Fusarium*-contaminated diet showed a significant upregulation of xanthine oxidoreductase (XOR); interferon gamma (IFNg); interleukins (IL)-8, -10, and -12; PEPT1; and claudin 1 (CLDN1). This effect was no longer observed when the *Fusarium*-contaminated diet was supplemented with yeast β-glucan (Figure 2).

The expressions of the evaluated markers in the liver such as heme oxygenase (HMOX), XOR, IFNg, IL-8, IL-10, IL-12, carnitine palmitoyltransferase 1 (CPT1), LEAP2, and sterol regulatory element binding transcription factor 2 (SREBP2) were not affected by the dietary treatments, except by a significant upregulation of inducible nitric oxide synthase (iNOS) (1.2-fold increase) in the liver of the broiler chickens fed the *Fusarium*-contaminated diet, and this effect was not observed when the *Fusarium*-contaminated diet was supplemented with yeast β-glucan. Furthermore, the expression of IL-6 in the liver was negligible.

### 2.4. Bacterial Translocation

**Figure 3:**
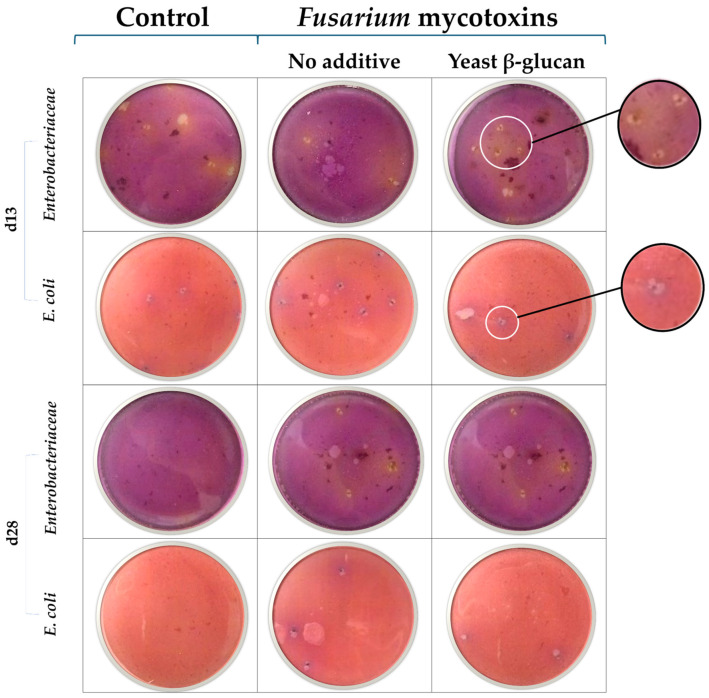
Representative images of Petri films specific for *E. coli* and *Enterobacteriaceae* after 24 h in vitro culture of liver samples from broiler chickens after being fed different experimental diets for 13 or 28 days. Inserts show typical colonies of *Enterobacteriaceae* (regularly shaped gas colonies) and *E. coli* (regularly shaped blue colony with gas).

No differences in bacterial colony counts were observed on day 13. On day 28, broiler chickens fed the *Fusarium*-contaminated diet showed a significant increase in the colonies of *Escherichia coli* in the liver. When yeast β-glucan was added to the *Fusarium*-contaminated diet, the number of *E. coli* colonies was similar to that in the control group (Table 4). The translocation of *Enterobacteriaceae* and *E. coli* to the liver was characterized by regularly shaped gas colonies (Figure 3). Irregularly shaped gas areas or bubbles were considered artifacts.

### 2.5. Macroscopic Findings

**Figure 4:**
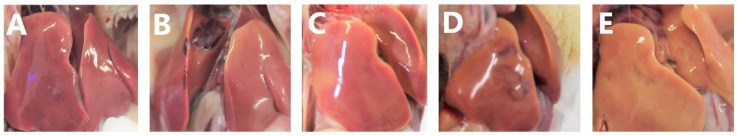
Representative images of normal liver (**A**), liver with signs of bleeding (**B**), pale areas (**C**), combination of bleeding and pale areas (**D**), and pale liver (**E**).

The post mortem analysis revealed some alterations in the liver (Figure 4). On both days 13 and 28, the group fed the *Fusarium*-contaminated diet had a higher number of broiler chickens displaying blood spots or pale areas in their livers. Table 5 depicts the number of broiler chickens exhibiting alterations and the total number of evaluated broiler chickens per treatment, i.e., 18 (six cages with three birds per cage). A sum of the number of broiler chickens presenting alterations higher than the total number of chickens indicates that the same chicken presented two or more alterations. 

## 3. Discussion

In the present study, we evaluated the effects of a diet naturally contaminated with *Fusarium* mycotoxins, where the main mycotoxin was DON at a concentration of approximately 3 mg/kg. The dietary levels of ZEN and FUM were extremely low when considering the LOAEL of 30 mg/kg BW per day and 2.5 mg/kg feed, respectively [9,21]. The levels of 3+15-ADON and DON-3G were comparable in the contaminated diets. Furthermore, different from pigs, the oral bioavailability of DON-3G in poultry is very low (3.8%), and presystemic hydrolysis and conversion into DON do not occur [22]. Therefore, the results are discussed regarding the effects of DON exposure on growth performance, intestinal and liver function, and bacterial translocation in broiler chickens. Furthermore, the impact of yeast β-glucan supplementation of the *Fusarium*-contaminated diet was evaluated.

Although broiler chickens fed the *Fusarium*-contaminated diet had a BWG 23 g lower than those fed the control diet, no significant differences were observed in growth performance. The trial was performed under highly controlled climate conditions, and the birds were kept in cages and not in floor pens like in other studies [8]. Impaired growth performance was reported in broiler chickens exposed to 2.3 mg/kg DON via a wheat-based diet, which is a source of nonstarch polysaccharides (NSPs), responsible for increasing intestinal viscosity in poultry and acting as an extra dietary challenge [5]. In the present trial, birds were housed in low density, and they were not submitted to the same risks observed in a commercial large farm, e.g., extra viral and bacterial exposure. When performance was assessed in 18 trials in a longitudinal study performed in commercial poultry farms, a significant loss in growth performance was observed when the dietary DON levels were close to 2.6 mg/kg [4].

Regarding the morphological alterations in the intestines, at d13, the VH and CD of the jejunum were significantly decreased when broiler chickens were fed the *Fusarium*-contaminated diet, except when yeast β-glucan was added to the diet. This effect was expected and already observed in previous trials with broiler chickens [6,23]. Deoxynivalenol induces apoptosis and inhibits cell proliferation [7]. The VH:CD was increased in the jejunum of 28-day-old chickens fed the *Fusarium*-contaminated diet supplemented with yeast β-glucan because of an increase in the villus height. These findings are in agreement with previous research, which showed that birds fed β-glucans had increased villus height [24,25] but also indicated that the yeast β-glucan can help recover some of the villus loss or damage caused by *Fusarium* challenge. It was observed that yeast β-glucans enhanced intestinal health by increasing immune-response-stimulating macrophages [25]. Furthermore, dietary supplementation with yeast β-glucans decreased the intestinal lesions caused by necrotic enteritis, demonstrating its healing capacity [26]. Exposure to *Fusarium* mycotoxins during 28 days decreased the ileum villus area, but neither the villus height nor the crypt depth changed along with this decrease. The decrease in villi surface area could be attributed to a reduction in their density, the atrophy of microvilli, changes in their shape, or tissue damage, even if their height and the depth of the crypts remained unchanged. No increase in the degree of intestinal damage in relation to the control was observed, but it was remarkable that, at d28, the damage degree in all groups was above two. The trial was performed during the summer, and between d20 and d28, the temperature in the house was higher than the target, probably resulting in heat stress. As previously demonstrated, the jejunum is more sensitive to heat stress than the ileum [27], so no increase in damage was observed in the ileum during the present trial. The absence of significant differences among the treatments suggests that this period of heat stress did not increase the negative impact of the mycotoxins or increase mortality. None of the selected markers were altered in the liver, except for the upregulation of iNOS in the liver of 13-day-old broiler chickens. Exposure to DON may activate proinflammatory-inducible enzymes like iNOS at the site of inflammation [28]. However, no other cytokine was activated in the liver, indicating that this process was limited, and the broiler chickens were able to adapt to the condition of chronic exposure to mycotoxins on day 28.

Most of the alterations in gene expression were observed in the jejunum of the broiler chickens. On d13, broiler chickens fed the *Fusarium*-contaminated diet showed a downregulation of PEPT1, an upregulation of GLUT1, and an upregulation of LEAP2. Previous studies have reported the downregulation of PEPT1 in the jejunum of broiler chickens fed a DON-contaminated diet [29,30]. PEPT1 is expressed in the brush border membrane of enterocytes and plays a role in the transport of most amino acids [31,32]. The downregulation of PEPT1 is the result of an adaptation mechanism due to the decreased protein use in the first two weeks of dietary exposure to DON or to a direct negative impact of DON, which decreases brush border function [33]. This later hypothesis is supported by the decreased villus height in the present trial, consequently interfering with the nutrient absorption area. PEPT1 downregulation was concomitant with GLUT1 upregulation, which may act as a compensatory response [6] because this glucose transporter is essential for cellular growth and development [34]. In the present trial, the antimicrobial peptide LEAP2 was upregulated in the jejunum of 13-day-old broiler chickens fed the DON-contaminated diet. The expression of LEAP2 can be upregulated as a response to bacterial and protozoan infections as a direct systemic response [35,36]. When the contaminated diet was supplemented with yeast β-glucan, GLUT1 upregulation remained present, but no downregulation of PEPT1 was observed.

The broiler chickens fed the DON-contaminated diet showed an upregulation of PEPT1 in their jejunum on d28, potentially as a response to the decreased nutrient absorption caused by the long-term exposure to DON. The 28-day exposure resulted in oxidative stress and inflammation, as measured by the upregulation of XOR, IFNg, IL8, IL10, and IL12. The upregulation of XOR in the intestine of broiler chickens fed a DON-contaminated diet was previously shown [8]. This enzyme plays a role in the synthesis of reactive oxygen species as a cellular defence [37]. DON has proinflammatory properties and causes persistent intestinal inflammation [38]. Chronic inflammation caused by DON negatively impacts the immune system and decreases disease resistance in poultry [39]. It is known that β-glucans are not able to degrade or bind DON but act to improve gut health and bird immunity [40]. In the present study, it seems that yeast β-glucan was able to counteract jejunum inflammation. Supplementation with β-glucan has been demonstrated to modify the cytokine profiles of broiler chickens [41]. Exposure to high levels of DON, i.e., 10 mg/kg, resulted in the downregulation of the tight junction CLDN1 [42]. In the present trial, exposure to 3 mg/kg DON resulted in the upregulation of CLDN1. Disease conditions, including intestinal inflammation, likely increase the expression of this tight junction complex protein as a protective reaction [43]. The increase in the expression of CLDN1, however, did not prevent *E. coli* translocation in 28-day-old chickens fed the DON-contaminated diet. DON-facilitated bacterial translocation was demonstrated in in vitro studies with intestinal organoids [44] and in broiler chickens coexposed to DON and infectious agents [10]. The exposure of broiler chickens via their diet to 5 mg/kg DON resulted in an increase in the load of *E. coli* in the liver [9], as confirmed by our present findings. In the present trial, infection was not induced, and management was performed under strict hygienic conditions.

## 4. Conclusions

Exposure to a diet naturally contaminated with *Fusarium* mycotoxins, where the major contaminant was DON at a concentration of approximately 3 mg/kg, impaired jejunum villus height, crypt depth, and villus area as well as led to intestinal oxidative stress and inflammation with bacterial translocation. Supplementing the contaminated diet with yeast β-glucan counteracted this negative impact.

## 5. Materials and Methods

### 5.1. Experimental Design

One-day-old male Ross 308 broilers were purchased from a local commercial hatchery and divided into three experimental groups of 234 chicks each (divided among six replicate cages with 13 chicks each). The birds were housed in 18 cages with wood shavings as the bedding material and were kept until 28 days of age. Housing and management were performed following EU legislation [6]. The control diet was prepared with a maize batch marginally contaminated with mycotoxins. The other two diets were prepared with a maize batch naturally contaminated with *Fusarium* mycotoxins. All diets met the nutritional requirements of broiler chickens (Table 6). The contaminated diets were divided into two sub-batches, and one of them was supplemented with a yeast β-glucan (enriched yeast β-glucan, Phileo by Lesaffre, Marcq-en-Baroeul, France) at a dosage of 125 mg/kg diet. Treatments were randomly allocated per block to cages, where each treatment was repeated six times. The cage was the experimental unit, and each cage contained 13 broilers. On d13 and d28, three broiler chickens per cage were randomly selected and euthanized using gasification followed by exsanguination. Subsequently, jejunum and ileum samples from each of the three birds were immediately collected and prepared for histological analysis, while jejunum and liver samples were processed for mRNA expression analysis. Additionally, liver samples were collected under sterile conditions and subjected to a bacterial translocation test. Simultaneously, a post mortem examination of the broiler chickens was conducted.

The mycotoxin levels in each diet are presented in Table 7. In brief, the control diets contained negligible amounts of the *Fusarium* mycotoxins DON, DON-3G, nivalenol, and ZEN. In the *Fusarium*-contaminated diets, DON was the major mycotoxin, followed by DON-3G, ZEN, with negligible levels of FB_1_ + FB_2_, and 3+15-ADON. All diets were analyzed in an independent and accredited (BELAC 057-TEST/ISO17025) laboratory (Primoris Holding, Gent, Belgium) via liquid chromatography with tandem mass spectrometry (LC-MS/MS).

The analyzed mycotoxins with their respective limit of quantification (LOQ) were aflatoxin B_1_ (1 µg/kg), aflatoxin B_2_ (1 µg/kg), aflatoxin G_1_ (1 µg/kg), aflatoxin G_2_ (1 µg/kg), alternariol (2 µg/kg), alternariol monomethyl ether (2 µg/kg), beauvericin (5 µg/kg), citrinin (10 µg/kg), cytochalasine E (2 µg/kg), deoxynivalenol (20 µg/kg), 3 + 15 acetyl-deoxynivalenol (3 + 15 ADON; 20 µg/kg), deoxynivalenol-3-glucoside (DON-3G; 20 µg/kg), diacetoxyscirpenol (5 µg/kg), enniatin A (5 µg/kg), enniatin A1 (5 µg/kg), enniatin B (5 µg/kg), enniatin B1 (5 µg/kg), fumonisins B_1_ + B_2_ (20 µg/kg), moniliformin (5 µg/kg), nivalenol (50 µg/kg), ochratoxin A (1 µg/kg), roquefortine C (5 µg/kg), sterigmatocystin (1 µg/kg), T-2/HT-2 toxin (10 µg/kg), and zearalenone (15 µg/kg).

### 5.2. Production Performance

Broilers were weighed per cage on d0, d13, and d28, and mortality was recorded throughout the experimental period. BWG, FI, and FCR were determined in the periods from d0 to 13 and d13 to 28, and the complete experimental period, i.e., d0-28.

### 5.3. Histological Analysis

Samples of the jejunum and ileum from each three of the birds per cage on d13 and d28 were collected and fixed in buffered formalin for histological analysis. In brief, histological slides (periodic acid–Schiff (PAS) counterstained with hematoxylin staining) from the jejunum were scanned with a NanoZoomer-XR (Hamamatsu Photonics KK, Hamamatsu, Japan). The scanned slides were viewed through viewer software (NDP.view2; Hamamatsu) and analyzed using analysis software (NDP.analyze; Hamamatsu). VH, CD, and villus area (µm^2^) from each individual bird were measured (15 villi per intestinal segment). The measurements of VH and CD were used to calculate the VH:CD ratio. Only intact villi were measured. To evaluate the degree of mucosal damage, the Chiu/Park scale was applied [45]. In brief, the mucosa was classified as normal if presenting an intact structure with no visible damage (degree 0) to severely damaged (degree 6) [27]. The mean damage degree per treatment was calculated, as previously described [46].

### 5.4. mRNA Expression of Markers of Gut Integrity and Liver Function

From each of the three birds per cage, samples of the jejunum and liver were collected for RNA isolation using an SV Total RNA Isolation System (Promega, Madison, WI, USA) according to the manufacturer’s instructions, and total RNA was quantified with a spectrophotometer (Nanodrop ND-1000, Thermo Scientific, Wilmington, DE, USA). Subsequently, 1 µg of extracted total RNA was reverse-transcribed with an iScriptTM cDNA Synthesis kit (BIO-RAD, Hercules, CA, USA). The cDNA was diluted to a final concentration of 30 ng/µL. Primers, as presented in Table 8, were commercially produced (Eurogentec, Maastricht, The Netherlands). qPCR was performed as previously described [6], and data were analyzed using the efficiency-corrected DeltaDelta-Ct method [47]. The fold-change values of the genes of interest were normalized using the geometric mean of the fold-change values of two housekeeping genes: hypoxanthine-guanine phosphoribosyl transferase (HPRT) and b-actin (ACTB). The mRNA expression of the markers in the jejunum and ileum were selected based on their role, i.e., oxidative stress, inflammation, nutrient transporters, villus crypt function, tight junctions, and intestinal damage. The markers in the liver were based on their role, i.e., oxidative stress, inflammation, and metabolism.

### 5.5. Bacterial Translocation

To evaluate the degree of bacteria that moved from the gut to the liver, bacterial translocation was determined. From each of the three birds per cage, the right half of the liver was removed, collected in sterile bags, and homogenized with sterile 0.9% saline [54]. After mixing the tissue well in saline, 1 mL of each sample was plated on two 3M Petri films (3M, Delft, The Netherlands) with specificity for *E. coli* and *Enterobacteriaceae*. Measurements of colony growth were performed after 24 h incubation at 37 °C. Translocation was determined by counting the number of bacterial colonies per plate.

### 5.6. Statistical Analysis

The cage was the experimental unit for all data. The experimental data were analyzed with ANOVA (GenStat Version 19.0, 2018, Hemel Hempstead, UK). Treatment means were compared using the least significant difference (LSD). Values with *p* ≤ 0.05 were considered statistically significant.

### 5.7. Declaration of AI-Assisted Technology in the Writing Process

During the preparation of this work, the authors used QuillBot in order to improve readability and language. The AI tool was not used to replace key researcher tasks such as interpreting data or drawing scientific conclusions. After using this tool, the authors reviewed and edited the content as needed and take full responsibility for the content of this publication.

## Figures and Tables

**Table 1:** Mean BW (g), BWG (g), FI (g), FCR (g/g), and mortality rate (%) of broiler chickens fed the experimental diets.

	Control	*Fusarium* Mycotoxins		*p*-Value	LSD
		No Additive	Yeast β-Glucan		
**d0-13**					
BW d13	382	385	389	0.81	23.2
BWG (g)	339	341	356	0.22	20.9
FI (g)	417	415	424	0.54	18.3
FCR (g/g)	1.232	1.218	1.193	0.49	0.0681
Mortality (%)	1.3	0.0	1.3	NA	NA
**d13-28**					
BW d28	1322	1319	1344	0.72	69.6
BWG (g)	928	937	955	0.61	57.2
FI (g)	1338	1329	1389	0.12	61.0
FCR (g/g)	1.442	1.420	1.455	0.28	0.0454
Mortality (%)	1.3	0.0	0.0	NA	NA
**d0-28**					
BWG (g)	1279	1256	1307	0.28	64.8
FI (g)	1776	1751	1819	0.26	85.0
FCR (g/g)	1.389	1.394	1.392	0.97	0.0376
Mortality (%)	2.6	0.0	1.3	0.14	2.94

BW: body weight; BWG: body weight gain; FI: feed intake; FCR: feed conversion ratio; NA: not applicable.

**Table 2:** Mean villus height (µm), crypt depth (µm), VH:CD (µm:µm), villus area (mm^2^), and damage score in the jejunum of the broiler chickens fed the experimental diets.

	Control	*Fusarium* Mycotoxins		*p*-Value	LSD
		No Additive	Yeast β-Glucan		
**d13**					
Villus height (µm)	826 ^b^	742 ^a^	795 ^ab^	0.048	66.3
Crypt depth (µm)	192 ^b^	159 ^a^	203 ^b^	<0.001	14.1
VH:CD	4.6	4.2	4.2	0.38	0.67
Villus area (mm^2^)	0.13	0.08	0.12	0.07	0.040
Score	0.16	0.31	0.12	0.47	0.342
**d28**					
Villus height (µm)	822	926	951	0.48	235.5
Crypt depth (µm)	238	231	229	0.94	55.5
VH:CD	3.6 ^a^	4.1 ^ab^	4.5 ^b^	0.02	0.58
Villus area (mm^2^)	0.16	0.12	0.14	0.57	0.080
Score	2.53	2.86	2.37	0.63	1.086

^a,b^ Values without a common letter within a column differ significantly (*p* < 0.05).

**Table 3:** Mean villus height (µm), crypt depth (µm), VH:CD (µm:µm), villus area (mm^2^), and damage score in the ileum of the broiler chickens fed the experimental diets.

	Control	*Fusarium* Mycotoxins		*p*-Value	LSD
		No Additive	Yeast β-Glucan		
**d13**					
Villus height (µm)	469	522	520	0.13	58.0
Crypt depth (µm)	163	172	167	0.81	0.40
VH:CD	2.9	3.2	3.3	0.14	0.40
Villus area (mm^2^)	0.05	0.05	0.05	0.25	0.010
Score	1.43	1.52	1.19	0.16	0.358
**d28**					
Villus height (µm)	594	623	645	0.70	125.9
Crypt depth (µm)	192	193	202	0.76	31.2
VH:CD	3.4	3.3	3.3	0.87	0.47
Villus area (mm^2^)	0.13 ^b^	0.10 ^a^	0.08 ^a^	<0.001	0.020
Score	0.51	0.41	0.28	0.53	0.433

^a,b^ Values without a common letter within a column differ significantly (*p* < 0.05).

**Table 4:** The mean number of bacterial colonies recovered in the liver of the broiler chickens fed the experimental diets.

	Control	*Fusarium* Mycotoxins		*p*-Value	LSD
		No Additive	Yeast β-Glucan		
**d13**					
*Enterobacteriaceae*	2.81	3.11	3.22	0.97	4.287
*E. coli*	4.28	2.17	3.00	0.60	4.430
**d28**					
*Enterobacteriaceae*	0.61	2.11	0.93	0.48	2.686
*E. coli*	0.28 ^a^	3.87 ^b^	2.11 ^ab^	0.049	2.802

^a,b^ Values without a common letter within a column differ significantly (*p* < 0.05).

**Table 5:** Macroscopical alterations in the liver of the broiler chickens fed the experimental diets.

	Control	*Fusarium* Mycotoxins	
		No Additive	Yeast β-Glucan
**d13**			
Blood spots	2/18	10/18	4/18
Pale areas	1/18	10/18	1/18
Pale liver	0/18	3/18	0/18
Total	3/18	11/18	5/18
**d28**			
Blood spots	0/18	11/18	0/18
Pale areas	1/18	9/18	2/18
Pale liver	0/18	0/18	0/18
Total	1/18	11/18	2/18

**Table 6:** Composition of the experimental diets.

	Starter (d0-13)	Grower (d13-28)
**Ingredient (%)**		
Maize	45.00	45.00
Soybean meal	34.91	30.73
Wheat	13.79	17.39
Soybean oil	0.00	0.76
Poultry fat	2.79	3.00
Salt	0.33	0.24
Limestone	0.83	0.83
Monocalcium phosphate	1.26	0.89
Sodium bicarbonate	0.00	0.10
Lysine HCl	0.23	0.22
DL-methionine	0.30	0.27
Threonine	0.06	0.07
Valine	0.01	0.00
Vitamin and mineral premix	0.50	0.50
**Nutrients**		
Energy (kcal/kg)	2900	3000
DM, g/kg	878	878
Ash, g/kg	53.99	47.63
Crude protein, g/kg	222	206
Crude fat, g/kg	57.9	67.1
Crude fiber, g/kg	21.9	21.3
Ca, g/kg	6.46	5.72
P, g/kg	6.46	5.47
K, g/kg	9.69	8.92
Na, g/kg	1.40	1.30
Cl, g/kg	3.00	2.43

**Table 7:** Mycotoxin composition of the experimental diets.

Mycotoxin (mg/kg)	Control	DON	DON + Yeast β-Glucan
**d0-13**			
DON	0.136	3.38	3.64
3+15 ADON		0.042	0.046
DON-3G	0.022	0.550	0.610
Nivalenol	0.066		
Zearalenone	0.040	0.440	0.480
**d13-28**			
DON	0.089	3.24	3.34
3+15 ADON		0.065	0.061
DON-3G	0.023	0.570	0.820
Nivalenol	0.108		
FB_1_+FB_2_		0.077	0.113
Zearalenone		0.540	0.600

**Table 8:** Primers used for the quantification of gene of interest (GOI) and housekeeping gene (HKG) expression.

Genes	Primer Sequence	Annealing T°	Role	Reference
HKG				
HPRT	F:CGTTGCTGTCTCTACTTAAGCAG R:GATATCCCACACTTCGAGGAG	65	-	[46]
ACTB	F:ATGTGGATCAGCAAGCAGGAGTA R:TTTATGCGCATTTATGGGTTTTGT	61	-	[48]
GOI				
*Jejunum and liver*				
HMOX	F:CTTGGCACAAGGAGTGTTAAC R:CATCCTGCTTGTCCTCTCAC	63	Oxidative stress	[46]
iNOS	F:GGACAAGGGCCATTGCACCA R:TCCATCAGCGCTGCGCACAA	61	Oxidative stress	[36]
XOR	F:GTGTCGGTGTACAGGATACAGAC R:CCTTACTATGACAGCATCCAGTG	61	Oxidative stress	[46]
IFNg	F:CAAGCTCCCGATGAACGAC R:GCAATTGCATCTCCTCTGAGAC	64	Inflammation	[36]
IL-6	F:GCTCGCCGGCTTCGA R:GGTAGGTCTGAAAGGCGAACAG	59	Inflammation	[48]
IL-8	F:CACGTTCAGCGATTGAACTC R:GACTTCCACATTCTTGCAGTG	64	Inflammation	[36]
IL-10	F:CATGCTGCTGGGCCTGAA R:CGTCTCCTTGATCTGCTTGATG	60	Inflammation	[49]
IL-12	F:TCAAGGAGATGTAACCTGCAG R:CTTCGGCAAATGGACAGTAG	60	Inflammation	[50]
LEAP2	F:CTCAGCCAGGTGTACTGTGCTT R:CGTCATCCGCTTCAGTCTCA	65	Metabolism	[36]
GLUT	F:TTGCTGGCTTTGGGTTGTG R:GGAGGTTGAGGGCCAAAGTC	57	Nutrient transporters	[48]
PEPT1	F:CCCCTGAGGAGGATCACTGTT R:CAAAAGAGCAGCAGCAACGA	59	Nutrient transporters	[46]
CLDN1	F:CTGATTGCTTCCAACCAG R:CAGGTCAAACAGAGGTACAAG	58	Tight junctions	[46]
CLDN5	F:CATCACTTCTCCTTCGTCAGC R:GCACAAAGATCTCCCAGGTC	58	Tight junctions	[46]
MUC-2	F:ATGCGATGTTAACACAGGACTC R:GTGGAGCACAGCAGACTTTG	61	Intestinal damage	[51]
VIL-1	F:GGCACCAACGAGTACAACACCA R:CAATTGCATCTCCTCTGAGAC	61	Intestinal damage	[48]
*Liver*				
CPT-1	F:AAGGGTACAGCAAAGAAGATCCA R:CCACAGGTGTCCAACAATAGGAG	61	Metabolism	[52]
HMGCR	F:TTGGATAGAGGGAAGAGGGAAG R:CTCGTAGTTGTATTCGGTAA	61	Metabolism	[53]
SREBP2	F:CCCAGAACAGCAAGCAAGG R:GCGAGGACAGGAAAGAGAGTG	61	Metabolism	[53]

## Data Availability

The original contributions presented in this study are included in the article. Further inquiries can be directed to the corresponding author.

## Abbreviations

3-ADON	3-Acetyl-deoxynivalenol
15-DON	15-Acetyl-deoxynivalenol
ACTB	Beta actin
ANOVA	Analysis of variance
BW	Body weight
BWG	Body weight gain
CD	Crypt depth
CLDN1	Claudin 1
CLDN5	Claudin 5
CPT1	Carnitine palmitoyltransferase 1
DON	Deoxynivalenol
DON-3G	Deoxynivalenol-3-glucoside
EU	European union
FCR	Feed conversion ratio
FI	Feed intake
FUM	Fumonisin
GLUT1	Glucose transporter 1
GOI	Gene of interest
HKG	Housekeeping gene
HMGCR	3-Hydroxy-3-methylglutaryl-CoA reductase
HMOX	Heme oxygenase
HPRT	Hypoxanthine phosphoribosyltransferase
IFNg	Interferon gamma
IL-10	Interleukin 10
IL-12	Interleukin 12
IL-6	Interleukin 6
IL-8	Interleukin 8
iNOS	Inducible nitric oxide synthase
LC-MS/MS	Liquid chromatography with tandem mass spectrometry
LEAP2	Liver-expressed antimicrobial peptide 2
LOAEL	Lowest observed adverse effect level
LSD	Least significant difference
MUC2	Mucin 2
NOAEL	No observable adverse effect level
PAS	Periodic acid–Schiff
PEPT1	Peptide transporter 1
qRT-PCR	Quantitative reverse-transcription polymerase chain reaction
SREBP2	Sterol regulatory element binding transcription factor 2
VH	Villus height
VH:CD ratio	Villus height:crypt depth ratio
VIL1	Villin 1
XOR	Xanthine oxidoreductase
ZEN	Zearalenone
